# Adaptive optics for a time-resolved Förster resonance energy transfer (FRET) and fluorescence lifetime imaging microscopy (FLIM) *in vivo*

**DOI:** 10.1364/OL.385950

**Published:** 2020-05-06

**Authors:** Simao Coelho, Simon P. Poland, Viviane Devauges, Simon M. Ameer-Beg

**Affiliations:** 1 Comprehensive Cancer Centre, School of Cancer and Pharmaceutical Sciences, Guy’s Campus, King’s College London, London, UK; 2 EMBL Australia Node in Single Molecule Science, School of Medical Sciences, University of New South Wales, Sydney, NSW, Australia

## Abstract

Förster resonance energy transfer (FRET) and fluorescence lifetime imaging (FLIM) have been coupled with multiphoton microscopy to image *in vivo* dynamics. However, the increase in optical aberrations as a function of depth significantly reduces the fluorescent signal, spatial resolution, and fluorescence lifetime accuracy. We present the development of a time-resolved FRET-FLIM imaging system with adaptive optics. We demonstrate the improvement of our adaptive optics (AO)-FRET-FLIM instrument over standard multiphoton FRET-FLIM imaging. We validate our approach using fixed cellular samples with FRET standards and *in vivo* with live imaging in a mouse kidney.

Multiphoton fluorescence microscopy has inherent 3D sectioning due to the nonlinear dependency of the excitation [1]. Multiphoton microscopy is routinely used for *in vivo* imaging due to low photo-damage, a high signal-to-noise ratio (SNR), and increased depth penetration. However, imaging though the various refractive indices present in tissue introduces optical aberrations. Optical aberrations result in a degradation of resolution, image brightness, and contrast [2]. Specifically, due to the nonlinear process of multiphoton excitation, optical aberrations broaden the focal spot within the sample and further degrade the SNR [3].

Aberrations due to the sample’s heterogeneity can be minimized using adaptive optics (AO) [4]. AO relies on introducing a dynamic optical correction element, such as a spatial light modulator (SLM), into the imaging path. The purpose is to introduce an equal but opposite distortion to the wavefront, therefore negating aberrations within the specimen [5–7]. The wavefront can be modeled as phase variations in the pupil of the imaging objective. By applying an accurate phase delay to the laser illumination, the refractive variations of the sample are minimized. To appropriately characterize aberrations, it is convenient to represent them as a series of orthogonal functions, such as Zernike polynomials. Zernike polynomials are regularly used due to their mathematical simplicity and low-order Zernike modes closely correspond to traditional aberration terms, such as astigmatism, coma, or spherical aberration [8].

AO imaging in microscopy can be divided in two main categories: direct sensing and indirect optimization [9]. Direct sensing approaches typically employ a wavefront sensor (i.e., Shack–Hartmann), which sections the wavefront into areas and measures the wavefront via an image shift [10]. Indirect methods (i.e., random search) consist of an optimization algorithm which improves on a feature of the sample (i.e., intensity) using either a zonal [11,12] or pre-determined modal combination [4,13] of phase corrective patterns.

The AO method we adopted was a direct sensing approach based on pupil segmentation [13]. The pupil-segmentation method is based on sequentially illuminating individual sections of the imaging objective’s pupil. A SLM, which is optically conjugate to the pupil of the objective, displays a grating pattern. The phase pattern is masked in turn, therefore only illuminating one sub-section of the pupil at a time. Refractive index variations that are specific to the illuminated section of the pupil result in a change in position of the image. The position for each sub-section across the entire pupil of the objective is determined and subtracted to that of the fully illuminated pupil. This results in a local slope for each segment, and a phase reconstruction algorithm determines the wavefront. The method is analogous to the Shack–Hartmann wavefront sensor, in which each area of the pupil is analyzed in turn without the use of a lenslet array.

In our approach, wavefront variations are determined by illuminating a square region that corresponds to 1/9 of the imaging pupil. We adopted a stepped overlapping approach that consists of translating the illuminated area vertically and horizontally by steps equal to half the length of the mask. This results in 25 regions, instead of nine. The overlapping regions provide intermediate slope contributions which increase the wavefront reconstruction accuracy. The overlapping approach also reduces the bias of modal fitting towards the periphery of the pupil [14]. The intermediate slopes contribute equally to the Zernike polynomial reconstruction algorithm. The reconstructed Zernike polynomial phase pattern is used to compensate for the aberrations. AO corrections are performed by adding the calculated pattern to the SLM.

**Fig. 1.**
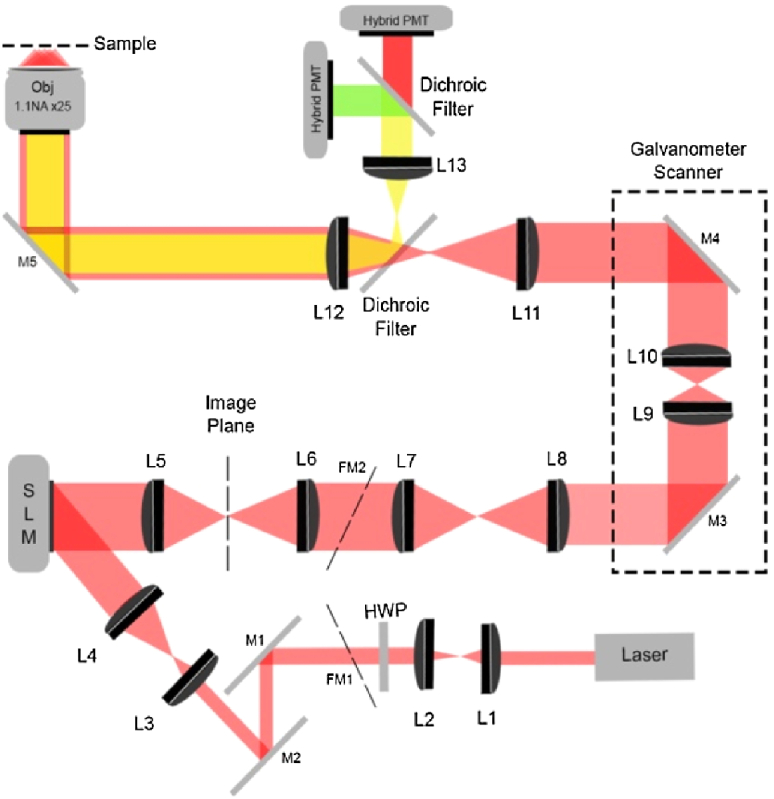
AO-FRET-FLIM system. A two-photon laser is expanded to overfill a SLM. The first-order diffraction pattern is filtered at the image plane of the SLM and relayed through a set of galvanometer scanners. The excitation is focused on the sample using a 1.1 NA objective. The sample fluorescence collected is spectrally split onto a pair of hybrid PMTs. L, lens; M, mirror.

In this Letter, we apply AO to multiphoton fluorescence lifetime imaging microscopy (FLIM). FLIM permits high spatiotemporal resolution imaging of dynamic processes, including Förster resonance energy transfer (FRET). Many questions related to cellular dynamics, such as protein interactions and conformational changes, can be addressed by FRET-FLIM [15–17]. To achieve high temporal precision, we used time-correlated single-photon counting (TCSPC). TSCPC permits picosecond time resolution conferring the most accurate lifetime determination [18].

The AO-FRET-FLIM optical layout is shown in Fig. 1. Laser light from a Ti:sapphire laser system (Spectra-Physics, DeepSee Mai Tai) was expanded to overfill a phase-domain SLM (Holoeye Photonics AG, Pluto-2 NIR). The SLM displays an eight-level blazed grating which efficiently projects the illumination pattern into the first order. The remaining orders are removed using an aperture in the image plane of the SLM. The SLM pattern is conjugated to the back pupil of a ×25 1.1 water dipping NA objective (Nikon Instruments, Ltd.) via a xy galvanometer scanning system (VM1000C, Cambridge Technology, Ltd.). Fluorescence is collected by the same objective lens, separated from the excitation using a dichroic (Semrock, Inc., FF670-SDi01), spectrally split using a second dichroic (Semrock, Inc., FF560-FDi02), and imaged onto two separate hybrid photomultiplier tubes (PMT) (Becker & Hickl GmbH, PMH-100) capable of TCSPC. Fluorescence is acquired in the non-descanned detection path. In contrast to a confocal microscope, a multiphoton AO microscope requires that the corrective optical element is placed only in the excitation optical path [9].

To determine aberrations induced by the optical components of the microscope, known as system aberrations, we imaged isolated fluorescent microspheres deposited on a cover slide. Imaging of red (emission >561nm) 0.2 µm diameter beads show a full width at half-maximum (FWHM) of 0.65±0.2 and 1.85±0.1µ, in the lateral and axial directions, respectively (Fig. 2). Performing AO correction reduces the FWHM to 0.56±0.02 and 1.1±0.07µm, in the lateral and axial directions, respectively. In addition, we demonstrate a ×1.6 increase in signal under the same experimental conditions. The amplitude of the various Zernike polynomials determined shows that the AO pattern primarily corrects for astigmatism, a common deformation of a SLM microdisplay [19]. The optical system corrective phase pattern is then added to the initial SLM blazed grating. This constitutes a pre-compensation for the optical train, and further experiments shown include this correction.

**Fig. 2.**
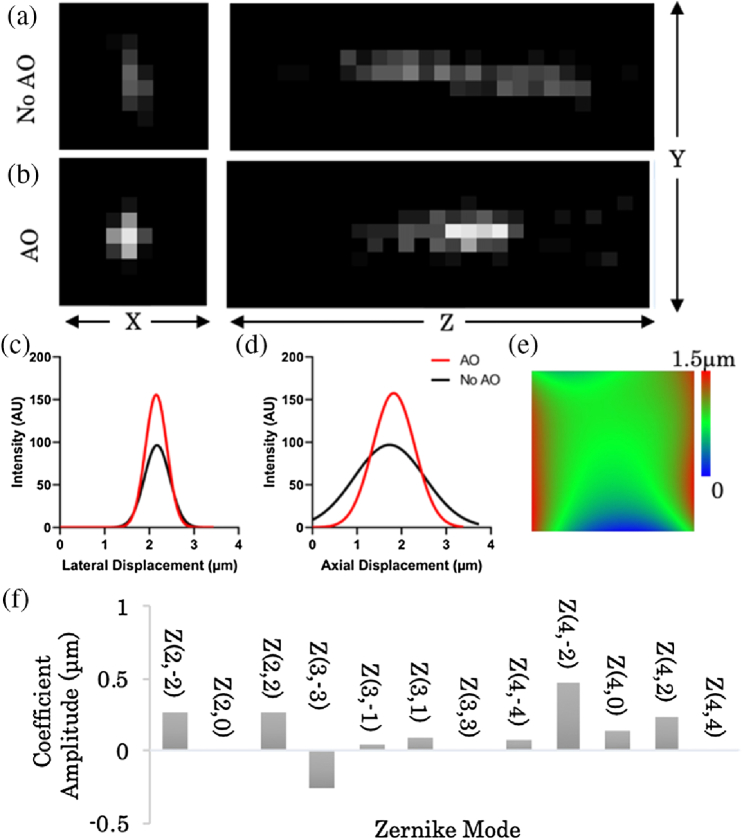
Correcting for system aberrations. Fluorescent microspheres with a 0.2 µm diameter were deposited onto a glass cover slide and imaged. Lateral and axial projections of (a) an uncorrected and (b) a system corrected microsphere. The average (c) lateral and (d) axial profiles of 20 separate beads before (black) and after (red) AO correction. (e) Corrective phase pattern. (f) Zernike modes and coefficients used in (e).

To evaluate the lifetime imaging performance of our AO imaging system for time-resolved FRET-FLIM, we used enhanced green fluorescence protein (EGFP) expressing cells and FRET standards [20]. Our FRET standards consist of two fluorescent proteins (EGFP and mRFP1) linked by a 7 amino acid linker, and thereby demonstrating high FRET efficiency. The FRET standards were expressed in HEK293 cells and placed under 50 µm mouse liver tissue slices (Fig. 3). The cells were transfected using Effectene Transfection Reagent (Qiagen, Ltd.) and, after 24 h, fixed in 4% paraformaldehyde. Fixed cells were placed on an imaging slide and dried in a laminar hood. A mouse liver of a C57BL/6 (B6) mouse was prepared by fixation in 4% paraformaldehyde for 24 hours, dehydrated in 30% sucrose for an additional 24 hours and subsequently frozen in optimal cutting temperature compound (Leica Biosystems). Cryostat sections of 50 µm thickness were placed on top of the cells and sealed under a coverslip with Vectra-Shield mounting media (Vector Laboratories, Inc.).

**Fig. 3.**
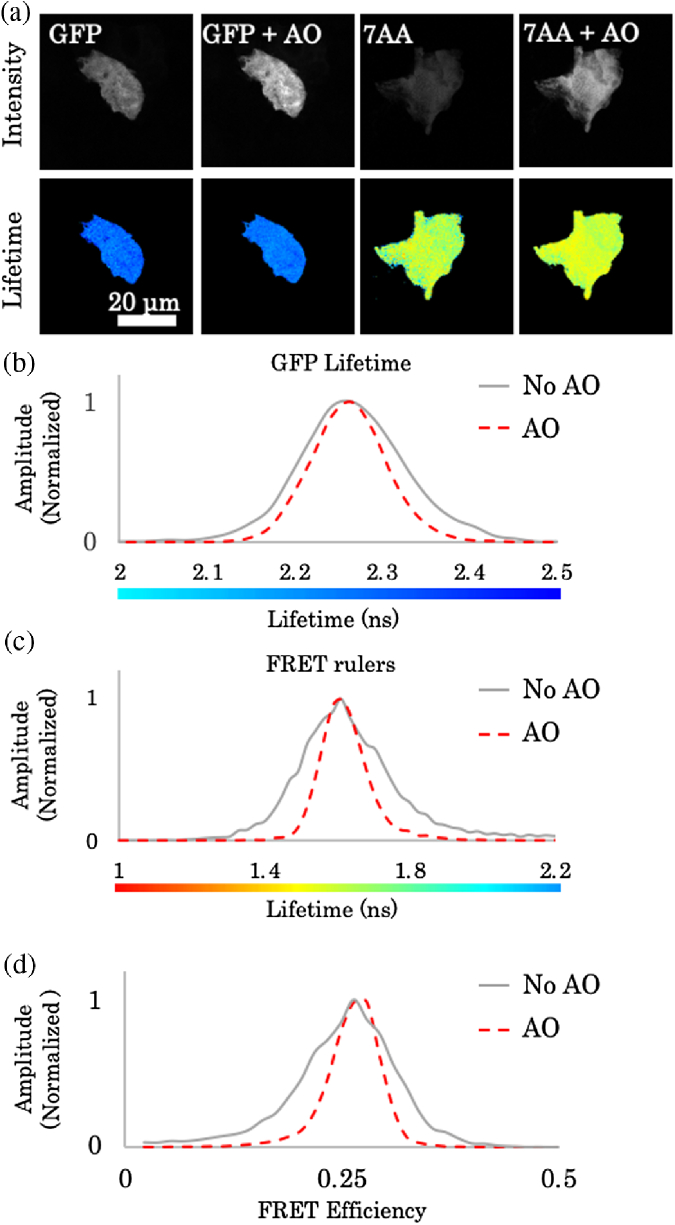
AO applied to FRET-FLIM. Multiphoton intensity and lifetime of HEK293 cells transfected with GFP (control) and 7AA FRET rulers. The cells were imaged under mouse liver sections with 50 µm thickness. (a) Fluorescence intensity and lifetime images using system aberration correction imaging and AO correction. (b) Normalized lifetime histogram of GFP control cells using standard imaging (gray) and AO (red). (c) Normalized lifetime histogram of 7AA cells using standard imaging (gray curve) and AO (red). (d) Normalized FRET histogram of 7AA FRET rulers using standard imaging (gray curve) and AO (red).

The mouse liver tissue placed between the cells and the objective introduces refractive index mismatches, which reduces the fluorescent intensity and broadens the FLIM histograms (Fig. 3). Performing AO correction reduces the influence of aberrations and demonstrates an increased accuracy of determining FRET-FLIM [Figs. 3(b)–3(d)]. This is evidenced by a reduced spread of the histogram. The FWHM of the FRET histogram was reduced from 0.12 to 0.06 with AO correction, a ×2 improvement. FRET efficiencies were calculated as $E\;=\;1-\tau /\tau_{D}$, where τ is the fluorescence lifetime of the probe in the presence of an acceptor and $\tau_{D}$ the control donor lifetime. The lifetime data were analyzed with 5×5 binning and Levenberg–Marquardt fitting in TRI2 lifetime analysis software. We determined the lifetime values of green fluorescence protein (GFP) to be 2.3±0.06 and 2.27±0.03ns with AO correction. The lifetime values of the FRET standards were determined to be 1.67±0.2 and 1.59±0.07ns with AO correction. The FRET efficiencies of the ruler constructs were determined as 27±6.8% and 29.7±3.3% with AO correction. These values are equivalent to previously reported values [20].

To assess the benefit of AO to image FRET-FLIM of fine structures in highly aberrating media, we imaged a fixed mouse liver stained with Alexa-488 at a depth of 100 µm (Fig. 4). Figure 4 demonstrates the potential of AO-FLIM to detect protein conformations in highly aberrated environments. Figure 4(a) shows a side-by-side comparison between FLIM images acquired for 300 s without AO and an AO corrected FLIM image acquired for 200 s. Thus, we also demonstrate that in highly aberrated environments our AO-FLIM is a significant improvement over prolonged acquisitions. The improvement in FLIM due to AO is highlighted in the zoomed-in area shown in Fig. 4(b). The highlighted feature would have been lost without AO. Figure 4(c) shows the lifetime histogram for Fig. 4(b) before and after AO correction. In Fig. 4(c), we demonstrate a ∼×4 improvement in the amplitude of the lifetime histogram counts and a ×1.5 reduction in standard deviation.

**Fig. 4.**
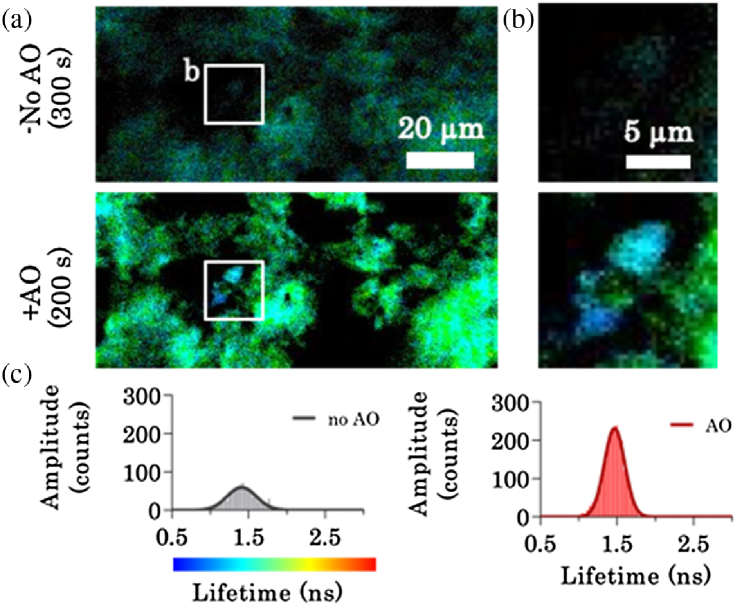
AO demonstrates increased precision in determining FLIM in highly aberrating media. (a) Combined images of multiphoton intensity and fluorescent lifetime. Increasing the acquisition time in highly aberrating media does not improve FLIM. Comparison of fluorescent lifetime after 300 s without AO and 200 s with AO. (b) Zoomed-in areas highlighted in (a) demonstrate fine structures which would have been lost without AO. (c) Fluorescent lifetime histogram of the area shown in (b). With AO, there is a ×4 increase in the lifetime histogram counts and a ×1.5 improvement in accuracy.

To evaluate the AO-FLIM capability in a challenging *in vivo* environment, we imaged macrophages within the kidney of a live mouse (Fig. 5). Cx3Cr1-GFP Rag2-/-Il2rg-/- mice were anaesthetized with a combination of ketamine (50 mg/kg), xylazine (10 mg/kg), and acepromazine (1.7 mg/kg) injected intraperitoneally. During imaging, anesthesia was maintained by the inhalation of 0.5% isoflurane in oxygen [21]. The kidney was surgically exposed, and the mouse was positioned on a custom-made stage insert with a glass coverslip. The temperature was maintained at 37°C using an environmental chamber.

**Fig. 5.**
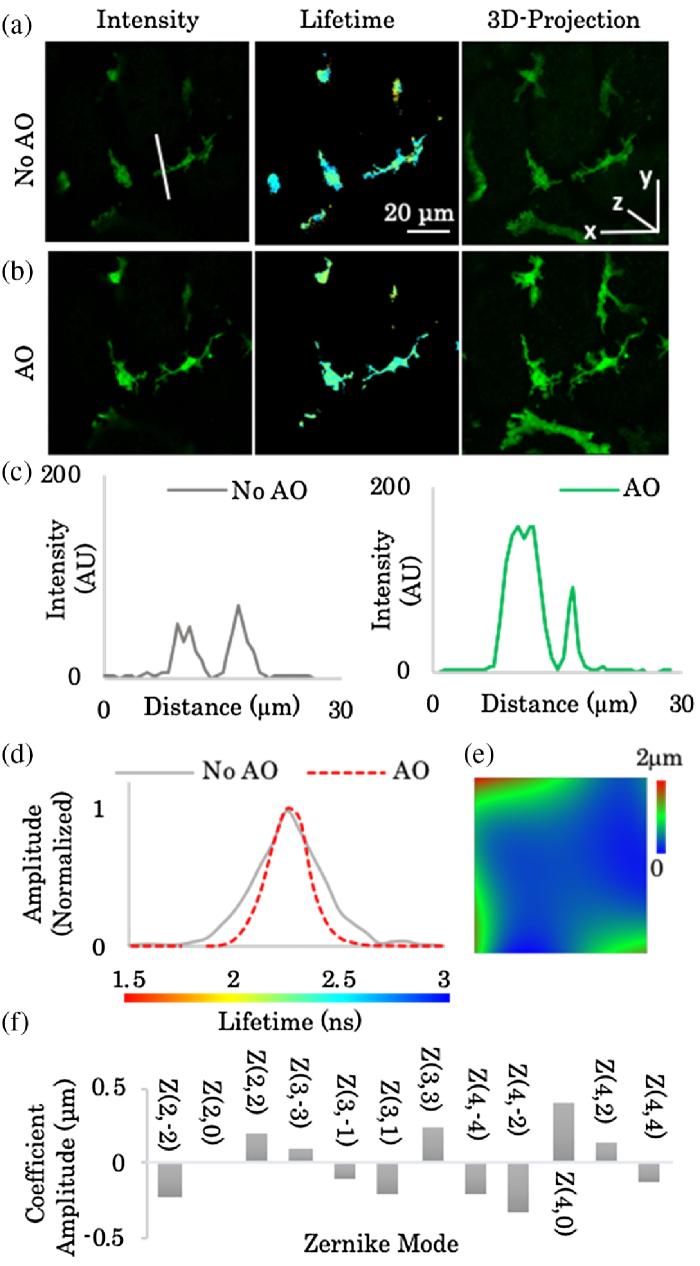
AO applied to FRET-FLIM *in vivo*. Intravital imaging of tissue resident macrophages in a live Cx3Cr1-GFP Rag2-/-Il2rg-/- mouse. Multiphoton intensity, lifetime, and Z-projection using (a) standard imaging and (b) AO correction. (c) Intensity line profile comparison between system corrected (No AO) and AO corrected shows a ×3 signal improvement. (d) Normalized FLIM histogram using standard imaging (gray curve) and AO correction (red curve). (e) Corrective phase pattern. (f) Zernike modes and coefficients used in (e).

We demonstrate a ∼3-fold improvement of the fluorescence intensity in three dimensions at a 200 µm depth over system corrected imaging [Figs. 5(a)–5(c)]. Normalized FLIM histograms also show an improvement due to AO correction [Figs. 5(d)–5(f)]. The FWHM of the lifetime histogram was reduced from 0.37 to 0.21 ns, an approximated ×1.8 improvement.

In conclusion, we demonstrate a proof-of-principle application of AO to two-photon FRET-FLIM microscopy. We demonstrate an improvement in fluorescence lifetime measurement uncertainty as a direct result of increasing both the resolution in aberrating media and photon count due to an improved Strehl ratio. We demonstrate improved FRET-FLIM imaging both *in vitro* using fixed cellular samples and *in vivo* by live mouse imaging.
